# Polypropylene/Short Glass Fibers Composites: Effects of Coupling Agents on Mechanical Properties, Thermal Behaviors, and Morphology

**DOI:** 10.3390/ma8125451

**Published:** 2015-12-02

**Authors:** Jia-Horng Lin, Chien-Lin Huang, Chi-Fan Liu, Chih-Kuang Chen, Zheng-Ian Lin, Ching-Wen Lou

**Affiliations:** 1 Laboratory of Fiber Application and Manufacturing, Department of Fiber and Composite Materials, Feng Chia University, Taichung City 40724, Taiwan; jhlin@fcu.edu.tw (J.-H.L.); chengyen0624@gmail.com (Z.-I.L.); 2 School of Chinese Medicine, China Medical University, Taichung City 40402, Taiwan; 3 Department of Fashion Design, Asia University, Taichung City 41354, Taiwan; 4 Department of Fiber and Composite Materials, Feng Chia University, Taichung City 40724, Taiwan; clhuang@mail.fcu.edu.tw; 5 Office of Physical Education and Sports Affairs, Feng Chia University, Taichung City 40724, Taiwan; cfliu@fcuoa.fcu.edu.tw; 6 The Polymeric Biomaterials Laboratory, Department of Fiber and Composite Materials, Feng Chia University, Taichung City 40724, Taiwan; chihkchen@fcu.edu.tw; 7 Institute of Biomedical Engineering and Materials Science, Central Taiwan University of Science and Technology, Taichung City 40601, Taiwan

**Keywords:** polypropylene (PP), Short glass fibers (SGF), coupling agent, mechanical properties, thermal behaviors

## Abstract

This study uses the melt compounding method to produce polypropylene (PP)/short glass fibers (SGF) composites. PP serves as matrix while SGF serves as reinforcement. Two coupling agents, maleic anhydride grafted polypropylene, (PP-g-MA) and maleic anhydride grafted styrene-ethylene-butylene-styrene block copolymer (SEBS-g-MA) are incorporated in the PP/SGF composites during the compounding process, in order to improve the interfacial adhesion and create diverse desired properties of the composites. According to the mechanical property evaluations, increasing PP-g-MA as a coupling agent provides the composites with higher tensile, flexural, and impact properties. In contrast, increasing SEBS-g-MA as a coupling agent provides the composites with decreasing tensile and flexural strengths, but also increasing impact strength. The DSC results indicate that using either PP-g-MA or SEBS-g-MA as the coupling agent increases the crystallization temperature. However, the melting temperature of PP barely changes. The spherulitic morphology results show that PP has a smaller spherulite size when it is processed with PP-g-MA or SEBS-g-MA as the coupling agent. The SEM results indicate that SGF is evenly distributed in PP matrices, but there are distinct voids between these two materials, indicating a poor interfacial adhesion. After PP-g-MA or SEBS-g-MA is incorporated, SGF can be encapsulated by PP, and the voids between them are fewer and indistinctive. This indicates that the coupling agents can effectively improve the interfacial compatibility between PP and SGF, and as a result improves the diverse properties of PP/SGF composites.

## 1. Introduction

Thermoplastic polymer has formed a new trend in material development, and it has the advantages of low production cost, great diversity, sufficient sources, light weight, good physical properties, and chemical resistance, as well as various efficient manufacturing process. Therefore, there are a great number of plastic products commercially available.

Polypropylene (PP) is the most consumed polymer globally, and it has a comparably light weight. Because of its good processing features, high chemical stability, and electrical insulation, PP has been commonly used in the spare parts of vehicles, bicycles, electronic products, civil necessities, medical instruments, and chemical products. However, it has low mechanical properties and does not have any functions, which restrict its application ranges [1,2,3,4,5]. Current studies reinforce PP in order to have greater mechanical properties; reinforcing materials include rigid particles fillers, such as nanoclay [6,7,8], calcium carbonate [9], and silicon dioxide [10,11], as well as short fibers, such as glass fiber, carbon fiber [12,13,14,15,16,17], and basalt fiber [18]. In particular, short glass fiber causes PP to be the strongest mechanically.

PP is a nonpolar polymer and has a poor interfacial adhesion with short glass fiber (SGF). This disadvantage is improved by using a coupling agent [19,20,21,22,23,24,25,26] or increasing the surface roughness of fibers during the compounding process [27,28,29]. Wong *et al.* examined three coupling agents for the mechanical properties and interfacial compatibilities of composites made of PP and recycled carbon fibers (CF). The test results showed that the tensile and flexural strengths of the composites were significantly improved. The interfacial compatibility of PP and CF was dependent on the acid anhydride group and molecular weight [30]. Broughton *et al.* used aminosilane and titanate as the coupling agent for glass flake and PP and found that the improved interfacial adhesion of the composites resulted in greater tensile, flexural, and impact strengths [31]. In contrast, Eslami-Farsani *et al.* added nanoclay to basalt fiber/polypropylene composite in order to increase the roughness of the fibers. The test results indicated that after the adhesion of nanoclay to the basalt fibers, the layered structure improved the interfacial adhesion of two materials, and thereby improved the mechanical properties of the composites [27]; however, the reinforcement was not permanent. In contrast, using a coupling agent can improve the interfacial adhesion between constituent materials, and this method forms a chemical reaction between the matrix and fibers, thereby effectively and permanently improving the mechanical properties of the composites.

This study aims to produce composites that meet the requirements of different products and to expand the applications of the composites, and compares different characterizations of the PP/SGF composites produced with two different coupling agents. In this study, short glass fibers that possess high strength, high modulus, and good thermal stability are synthesized with PP in order to reinforce the mechanical properties of PP. Two coupling agents (*i.e.*, maleic anhydride grafted polypropylene, PP-g-MA, and maleic anhydride grafted styrene-ethylene-butylene-styrene block copolymer, SEBS-g-MA) are used for a greater combination between PP and SGF. PP-g-MA has the same molecular structure that PP has, and thus enhances the bonding between two materials. Similarly, SEBS-g-MA, an elastomer, has a structure that includes an ethylene-butylene segment that is compatible with PP. Therefore, SEBS-g-MA can improve the compatibility between PP and SGF, and provides the composites with impact resistance. Finally, the influences of these coupling agents on the mechanical properties, thermal behaviors, spherulite structure, and interfacial adhesion are examined. This study can thus serve as a reference for the selection of a coupling agent according to the diverse applications of the composites.

## 2. Experimental Section

### 2.1. Materials

Polypropylene (PP; YUNGSOX 1080; Formosa Plastics Corporation, Taipei, Taiwan) was a homopolymer with a melt flow rate of 10 g/10 min (ISO1133). Short glass fiber (SGF; 202P; Taiwan Glass Ind. Corp., Taipei, Taiwan) had a length of 3.2 mm and a diameter of 13 µm, and was treated with a silane coupling agent. Maleic anhydride grafted polypropylene (PP-g-MA; DuPont Fusabond P613) was purchased from DuPont, Wilmington, DE, USA. Maleic anhydride grafted styrene-ethylene-butylene-styrene block copolymer (SEBS-g-MA; Kraton FG1901X) was purchased from Kraton, Houston, TX, US. Physical properties of materials are summarized in Table 1.

**Table 1:** Physical properties of materials. Polypropylene, PP; Short Glass Fiber, SGF; Maleic Anhydride grafted Polypropylene, PP-g-MA; Maleic Anhydride grafted Styrene-Ethylene-Butylene-Styrene block copolymer, SEBS-g-MA.

Material	Density (g/cm^3^)	Melt Index (g/10 min)	Diameter (µm)	Length (mm)	Graft Weight (%)
PP	0.900	10 (230 °C / 2.16 Kg measured)	-	-	-
SGF	-	-	13	3.2	-
PP-g-MA	0.903	120 (190 °C / 2.16 Kg calculated)	-	-	0.5
SEBS-g-MA	0.910	22 (230 °C / 5 Kg measured)	-	-	1.5

### 2.2. Methods

Various amounts of PP, a specified amount of 25 wt % of SGF, and 2, 4, 6, or 8 wt % of a coupling agent (PP-g-MA or SEBS-g-MA) were mixed to form different PP/SGF/PP-g-MA blends and PP/SGF/SEBS-g-MA blends. Different blends were dried in an oven at 80 °C for 8 h in order to remove moisture, after which they were made into PP/SGF/PP-g-MA pellets and PP/SGF/SEBS-g-MA pellets *via* a single screw extruder (SEVC-45, Re-Plast Extruder Corp., Miaoli, Taiwan), in which the temperatures of the three barrels and the die were 210 °C, 220 °C, 230 °C, and 210 °C, respectively, and the screw speed was 36 rpm.

The pellets were then dried in an oven at 80 °C for 8 h, followed by being made into composites by using an injection machine (Ve-80, VICTOR Taichung Machinery Works Co., Ltd., Taichung, Taiwan), in which the temperatures of three barrels and the nozzle were 210 °C, 220 °C, 230 °C, and 210 °C, respectively. The control group was the pure PP/SGF composites made of 25 wt % SGF and 75 wt % PP.

### 2.3. Measurements

#### 2.3.1. Tensile Tests

An Instron 5566 Universal Tester (Instron, Canton, MA, USA) was used to measure the tensile strength and tensile modulus, as specified in ASTM D638-10. Samples were made into dumbbell-shapes according to ASTM D638 Type IV. The crosshead speed was 5 mm/min. There were a total of 5 samples for each specification.

#### 2.3.2. Flexural Tests

The flexural test was performed by using an Instron 5566 Universal Tester (Instron, Canton, MA, USA), as specified in ASTM D790-10. The load and flexural modulus of the samples was measured. There were a total of 5 samples for each specification, and each sample had a size of 127 mm × 12.7 mm × 3.2 mm. The test speed was 2 mm/min, and the support span was 50 mm. The test results were then used to calculate the flexural strength with the following Equation (1):

(1) $$\sigma_{f_{max}}=\frac{3PL}{2bd^{2}}$$

where *P* is the load (N); *L* is the support span (mm); *b* is the sample width (mm); and *d* is the sample thickness (mm).

#### 2.3.3. Izod Impact Tests

This test followed ASTM D 256-10. An Izod impact strength tester (CPI, ATLAS, Mount Prospect, IL, USA) was used to measure the impact strength of the samples. The samples had a 45° V-shaped cut with a depth of 0.25 mm, and were sized as 63.5 mm × 12.7 mm × 3.2 mm. There were a total of 5 samples for each specification.

#### 2.3.4. Differential Scanning Calorimetry (DSC)

Composite samples of 8–10 mg were then placed in the DSC (Q200, TA Instruments, New Castle, DE, USA). Samples were heated from 40 °C to 200 °C at 10 °C/min increments, and were isothermally kept at 200 °C for 10 min, in order to delete the thermal history. Next, samples were cooled to 40 °C with the same increments. During the second cycle, the samples were heated and then cooled between these two temperatures with the same increments. The crystallinity of composites was calculated from Equation (2). The enthalpy corresponding to the melding of 100% crystalline PP is 209 J/g [32,33].

(2) $$X_{C}=\frac{\Delta H_{m}}{\Delta H_{m}^{{}^{\circ}}\times (1-f)}$$

where *X_C_* is crystallinity; *ΔH_m_* is the apparent enthalpy of crystallization; *ΔH°_m_* is the enthalpy corresponding to the melting of 100% crystalline PP; and *f* is the weight fraction of SGF.

#### 2.3.5. Polarized Light Microscopy (PLM)

The spherulite morphology of the samples was observed by using a PLM (BX51, Olympus, Tokyo, Japan). A small amount of sample was placed on a glass slide and was then melted to form a film at 200 °C. Samples were then cooled to 130 °C at 10 °C/min increments, and were kept at 130 °C for the observation of spherulite morphology.

#### 2.3.6. Scanning Electron Microscope (SEM)

Samples were affixed to the sample holder by using carbon paste and were then coated with a thin gold layer. The fractured surface of the samples was then observed by using an SEM (S3000N, Hitachi, Tokyo, Japan) at a voltage of 15 kV.

## 3. Results and Discussion

### 3.1. Effects of Two Coupling Agents (PP-g-MA or SEBS-g-MA) on Mechanical Properties of PP/SGF Composites

**Figure 1:**
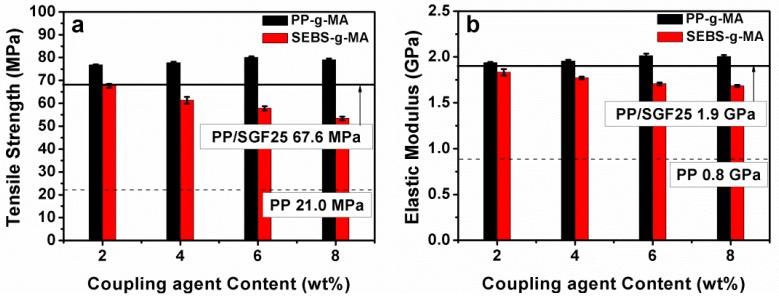
(**a**) Tensile strength and (**b**) tensile modulus of PP (Polypropylene)/SGF (short glass fiber) composites.

The tensile properties of various PP/SGF composites are compared to those of pure PP/SGF composites (*i.e.*, the control group), as indicated in Figure 1 and Table 2. The composites that are incorporated with 8 wt % PP-g-MA have a tensile strength of 79.0 MPa, in comparison to that of the control group (67.6 MPa). The tensile strength of the composites is proportional to the amount of PP-g-MA. In contrast, the composites that are incorporated with 8 wt % of SEBS-g-MA have a lower tensile strength (50.6 MPa) than that of the control group. The tensile strength is inversely proportional to the amount of SEBS-g-MA.

The tensile modulus of the PP/SGF composites incorporated with 8 wt % PP-g-MA is 2004 MPa, which is greater than that of the control group (1998 MPa). However, increasing PP-g-MA hardly influences the modulus of the composites. Conversely, the tensile modulus of the composites incorporated with 8 wt % SEB-g-MA is 1683.6 MPa, which is lower than that of the control group. Furthermore, the tensile modulus has a decreasing trend with increasing SEBS-g-MA.

**Figure 2:**
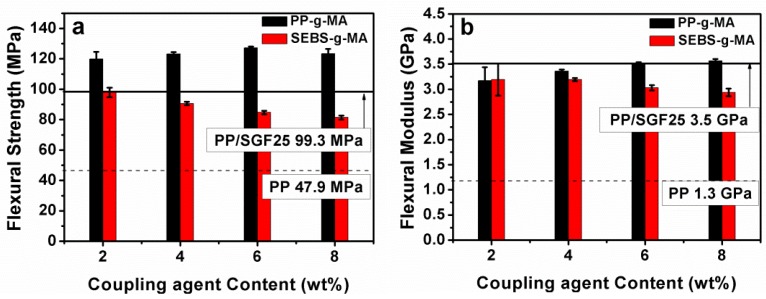
(**a**) Flexural strength and (**b**) flexural modulus of PP/SGF composites.

Figure 2 and Table 2 indicate the flexural properties of various PP/SGF composites that are in conjunction with different coupling agents. The incorporation of 8 wt % of PP-g-MA results in a greater flexural strength of the composites (123.4 MPa) in comparison to that of the control group (99.3 MPa). The flexural strength increases as a result of the increasing PP-g-MA. However, the incorporation of 8 wt % of SEBS-g-MA decreases the flexural strength of the composites to 81.3 MPa. Moreover, the flexural strength of the composites shows a declining trend with increasing SEBS-g-MA content.

**Table 2:** Mechanical properties of pure PP matrices and PP/SGF composites treated with various amounts of coupling agents.

Composite	σ_T_ (MPa)	E_T_ (GPa)	σ_F_ (MPa)	E_F_ (GPa)	IS (J/m)
**PP**	21.0 ± 0.8	0.8 ± 0.032	47.9 ± 1.3	1.3 ± 0.051	44.8 ± 0.0
**PP/SGF25**	67.6 ± 0.9	1.9 ± 0.026	99.3 ± 2.7	3.5 ± 0.045	73.7 ± 3.3
**PP-g-MA (2 wt %)**	76.8 ± 0.2	1.9 ± 0.008	120.0 ± 4.2	3.2 ± 0.268	101.9 ± 7.5
**PP-g-MA (4 wt %)**	77.7 ± 0.4	1.9 ± 0.013	123.3 ± 1.0	3.4 ± 0.031	103.5 ± 10.1
**PP-g-MA (6 wt %)**	80.0 ± 0.6	2.0 ± 0.022	127.2 ± 0.9	3.5 ± 0.032	103.5 ± 3.6
**PP-g-MA (8 wt %)**	79.0 ± 0.5	2.0 ± 0.016	123.4 ± 2.8	3.6 ± 0.035	97.5 ± 5.6
**SEBS-g-MA (2 wt %)**	61.3 ± 1.5	1.8 ± 0.035	97.8 ± 2.9	3.2 ± 0.320	77.7 ± 0.0
**SEBS-g-MA (4 wt %)**	57.8 ± 0.9	1.8 ± 0.014	90.6 ± 1.0	3.2 ± 0.031	82.0 ± 3.5
**SEBS-g-MA (6 wt %)**	53.4 ± 0.8	1.7 ± 0.016	84.8 ± 1.0	3.0 ± 0.052	86.2 ± 2.7
**SEBS-g-MA (8 wt %)**	50.6 ± 0.9	1.7 ± 0.012	81.4 ± 1.2	2.9 ± 0.076	90.3 ± 2.7

*Note.* σ_T_ is the tensile strength; E_T_ is the tensile modulus; σ_F_ is the flexural strength; E_F_ is the flexural modulus; and IS is the impact strength.

The flexural modulus of the PP/SGF composites increases to 3565.9 MPa as a result of the incorporation of 8 wt % PP-g-MA, in comparison to that of the control group (3503.4 MPa). The flexural strength does not fluctuate with increasing PP-g-MA. In contrast, the flexural modulus of the PP/SGF composites decreases to 2937.6 MPa, in comparison to the control group. The flexural modulus is inversely proportional to the content of SEBS-g-MA. 

**Figure 3:**
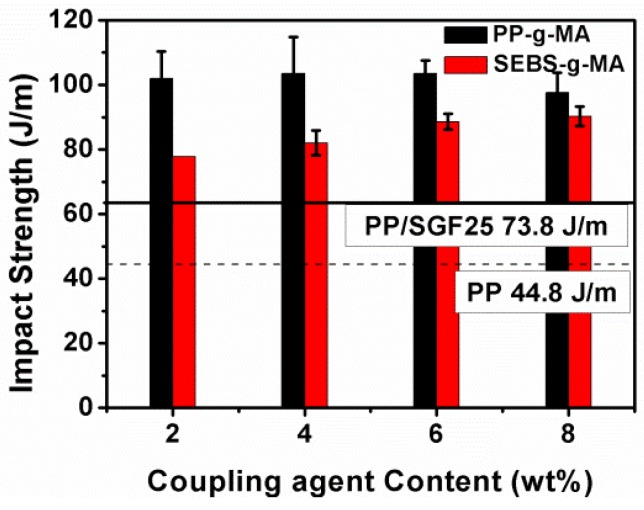
Impact strength of PP/SGF composites, as related to various coupling agents.

The impact strength of the PP/SGF composites that are incorporated with different coupling agents is indicated in Figure 3 and Table 2. The impact strength increases from 73.6 J/m to 97.5 J/m when the PP/SGF composites are incorporated with 8 wt % of PP-g-MA. Meanwhile, the impact strength increases from 73.6 J/m to 90.2 J/m when the PP/SGF composites are incorporated with 8 wt % of SEBS-g-MA. In summary, increasing coupling agent, either PP-g-MA or SEBS-g-MA, improves the impact strength of PP/SGF composites. Table 3 summarizes the variation in tensile, flexural, and impact properties as percentages.

**Figure 4:**
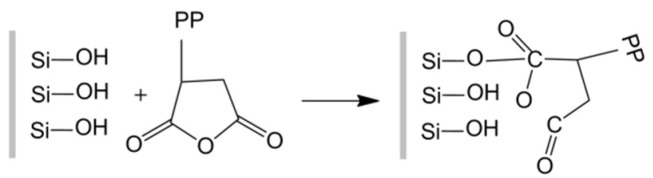
Chemical reaction between PP-g-MA and SGF.

The tensile, flexural, and impact strengths of PP/SGF composites are proportional to the content of PP-g-MA that is incorporated with the composites. The mechanical properties are reinforced due to the improved interfacial compatibility between PP and SGF. There is esterification between the hydroxyl groups on the surface of SGF and the acid anhydride groups of PP-g-MA, and the covalent bond is then formed, as indicated in Figure 4. As a result, PP-g-MA increases the interfacial compatibility between PP and SGF, and thereby improves the mechanical properties of the composites [30,34,35]. However, when PP-g-MA reaches 8 wt %, the aforementioned properties of the composites first exhibit an initial steep increase, followed by a gradual increase or even a steady decrease, which is ascribed to a saturated content of acid anhydride groups of PP-g-MA. In addition, the tensile modulus and flexural modulus of the composites barely change after the conjunction of PP-g-MA. There are two possible factors. SGF in PP/SGF composites is the major material that withstands deformation by an externally asserted force. PP-g-MA can only improve the interfacial compatibility between PP and SGF, but cannot help resist the deformation. Another factor is that PP-g-MA and PP have similar molecular weights. Therefore, the tensile modulus and flexural modulus of the composites are not correlated with the conjunction of PP-g-MA. Such a result is in line with the finding of the study by Wong *et al.* [30].

**Figure 5:**
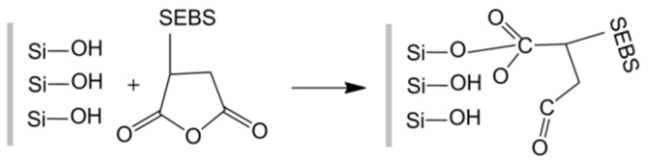
Chemical reaction between SEBS-g-MA and SGF.

The tensile and flexural strengths of PP/SGF composites decrease as a result of the combination of SEBS-g-MA. This coupling agent is an elastomer, which possesses low tensile and flexural strengths. A greater content of SEBS-g-MA causes the tensile and flexural strengths to decrease. However, the improved compatibility between SEBS-g-MA and SGF lends limited reinforcement to the tensile and flexural strengths of the composites. As indicated in Figure 5, the combination of SEBS-g-MA results in the chemical reaction between PP and SGF, which in turn improves their interfacial compatibility. This improved interfacial compatibility allows for elastic deformation of the composites under an impact, and the deformation can then absorb energy and toughen the composites, thereby increasing the impact strength. Although the toughness of the composite increases as a result of the combination of SEBS-g-MA, the tensile and flexural strengths of the composites decrease. Elastomers improve the toughness of the composites at the cost of sacrificing their rigidity and dimensional stability [36].

**Table 3:** Variations in tensile, flexural, and impact properties in percentage (%).

Coupling Agent (wt %)	PP-g-MA					SEBS-g-MA				
	σ_T_ (%)	E_T_ (%)	σ_F_ (%)	E_F_ (%)	IS (%)	σ_T_ (%)	E_T_ (%)	σ_F_ (%)	E_F_ (%)	IS (%)
**PP/SGF25**	0	0	0	0	0	0	0	0	0	0
**2 wt %**	14	−3	21	−9	38	−8	−8	−2	−8	5
**4 wt %**	15	−2	24	−4	41	−14	−11	−9	−8	12
**6 wt %**	18	1	28	1	41	−21	−14	−15	−13	17
**8 wt %**	18	0	24	2	33	−25	−15	−18	−16	23

### 3.2. Effects of Two Coupling Agents (PP-g-MA or SEBS-g-MA) on Thermal Behaviors of PP/SGF Composites

**Figure 6:**
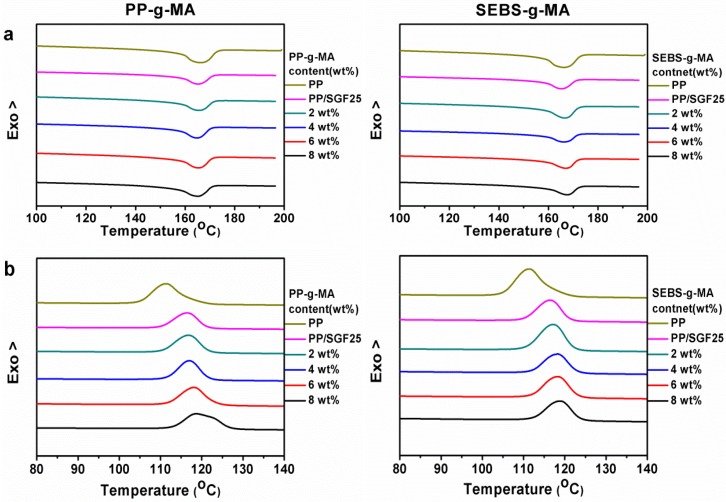
DSC curves for (**a**) melting behavior and (**b**) crystallization behavior of PP/SGF composites.

In comparison to the crystallization temperature (T_c_) and melting temperature (T_m_) of pure PP matrices and PP/SGF composites, PP/SGF composites that are incorporated with PP-g-MA have a slightly greater T_c_, but a similar T_m_, as indicated in Figure 6a,b, respectively.

The crystallization temperature of PP/SGF composites increases from 116.4 °C to 118.8 °C when the composites are incorporated with 8 wt % of PP-g-MA. For PP/SGF composites, SGF exists in PP, and is thus able to increase the crystallization temperature of PP. The subsequent combination of PP-g-MA enables SGF to be effectively distributed in and be compatible with PP. As a result, SGF has a greater surface area in PP matrices, which induced the nucleation of PP, and eventually augments the crystallization of PP [37].

The T_m_ of PP/SGF composites that are incorporated with PP-g-MA remains at 165.4 °C. Furthermore, the melting temperature does not pertain to the amount of PP-g-MA. Namely, the conjunction of PP-g-MA is not correlated with the crystal structure and thermal stability of PP.

Similarly, in comparison to the T_c_ and T_m_ of pure PP matrices and PP/SGF composites, the PP/SGF composites that are incorporated with SEBS-g-MA have a slightly greater T_c_ and a similar T_m_.

The T_c_ of PP/SGF composites increases from 116.4 °C to 118.8 °C when they are incorporated with 8 wt % of SEBS-g-MA. For PP/SGF composites, the T_c_ of PP increases as a result of the conjunction of SGF. SEBS-g-MA that is subsequently combined with the composites serves as a nucleating agent, which causes nucleation of PP and thus augments the T_c_ of PP/SGF composites. In addition, the T_m_ of the PP/SGF composites increases from 166.21 °C to 167.51 °C when the composites are incorporated with 8 wt % of SEBS-g-MA. According to the T_m_ results, the T_m_ of PP/SGF composites remains at 166.4 °C, which indicates that SEBS-g-MA does not correlate with the crystal structure and thermal stability of PP.

### 3.3. Effects of Two Coupling Agents (PP-g-MA or SEBS-g-MA) on Spherulite Morphology of PP/SGF Composites

**Figure 7:**
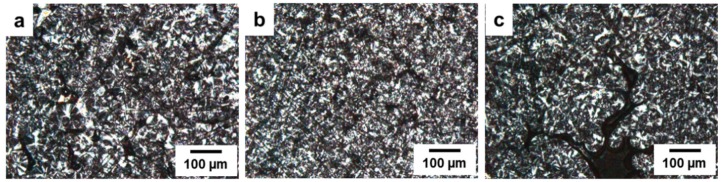
Polarized Light Microscopy images (100×) of the PP/SGF composites that are composed of (**a**) 0 wt % of a coupling agent; (**b**) 8 wt % of PP-g-MA as a coupling agent, and (**c**) 8 wt % of SEBS-g-MA as a coupling agent.

The PLM images of PP/SGF composites as related to various coupling agents are indicated in Figure 7. Figure 7a shows that when SGF is combined with PP, SGF serves as its nucleating agent. The distribution of SGF in PP matrices results in an increasing amount of spherulites, which in turn prevents the spherulites from being formed completely. As a result, the spherulite morphology is incomplete, and spherulites have a smaller size.

When 8 wt % of PP-g-MA or SEBS-g-MA is incorporated with SGF/PP composites, SGF is evenly distributed in PP matrices, as indicated in Figure 7b,c. A phenomenon that is similar to that observed in Figure 7a is found; namely, the spherulites have an incomplete structure and a smaller size with the identical reason addressed for Figure 7a [36,38].

**Figure 8:**
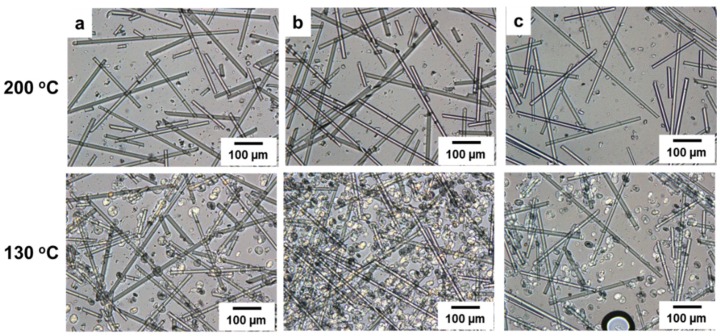
Optical microscopy images (100×) of the PP/SGF composites that are composed of (**a**) 0 wt % of a coupling agent; (**b**) 8 wt % of PP-g-MA as a coupling agent; and (**c**) 8 wt % of SEBS-g-MA as a coupling agent.

The optical microscopic images of PP/SGF composites that have been treated with different coupling agents are shown in Figure 8. PP is at a melting state when at a temperature of 200 °C, after which the majority of spherulites start to crystallize along the fibers at a temperature of 130 °C, which exemplifies that SGF is the nucleating agent for PP. Nevertheless, SGF is evenly distributed in PP as a result of the combination of PP, and thus there are more nucleating points created. The majority of PP’s spherulites are formed surrounding the fibers, and at the same time increases the crystallization temperature of PP, as indicated in Figure 6b and Table 4.

**Table 4:** Thermal behaviors of PP/SGF composites.

Coupling Agent (wt %)	PP-g-MA				SEBS-g-MA			
	Δ*H*_m_ (J/g)	*T*_m_ (°C)	*T*_c_ (°C)	*X*_c_ (%)	Δ*H*_m_ (J/g)	*T*_m_ (°C)	*T*_c_ (°C)	*X*_c_ (%)
**PP**	93.1	166.0	111.4	44.5	93.1	166.0	111.4	44.5
**PP/SGF25**	61.5	165.2	116.4	39.2	61.5	165.2	116.4	39.2
**2**	68.4	165.7	116.8	44.8	66.0	166.6	117.2	43.2
**4**	67.1	164.8	116.9	45.1	59.2	166.2	118.2	39.8
**6**	75.5	165.4	118.1	52.1	62.6	167.1	118.5	43.2
**8**	65.1	165.1	118.8	46.0	59.6	167.5	118.8	42.2

*Note.* ΔH_m_ is the melting enthalpy, T_m_ is the melting temperature, T_c_ is the crystallization temperature, and X_c_ is the crystallinity.

**Figure 9:**
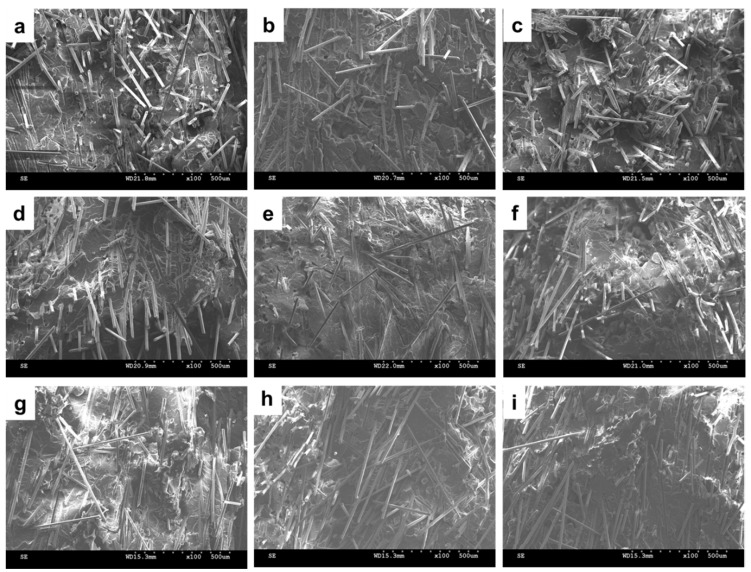
SEM images (100×) of PP/SGF composites, which are incorporated with (**a**) 0 wt % of a coupling agent; (**b**) 2 wt %; (**c**) 4 wt %, (**d**) 6 wt %; and (**e**) 8 wt % of PP-g-MA; as well as (**f**) 2 wt %; (**g**) 4 wt %; (**h**) 6 wt %; and (**i**) 8 wt % of SEBS-g-MA.

### 3.4. Effects of Two Coupling Agents (PP-g-MA or SEBS-g-MA) on Spherulite Morphology of PP/SGF Composites

**Figure 10:**
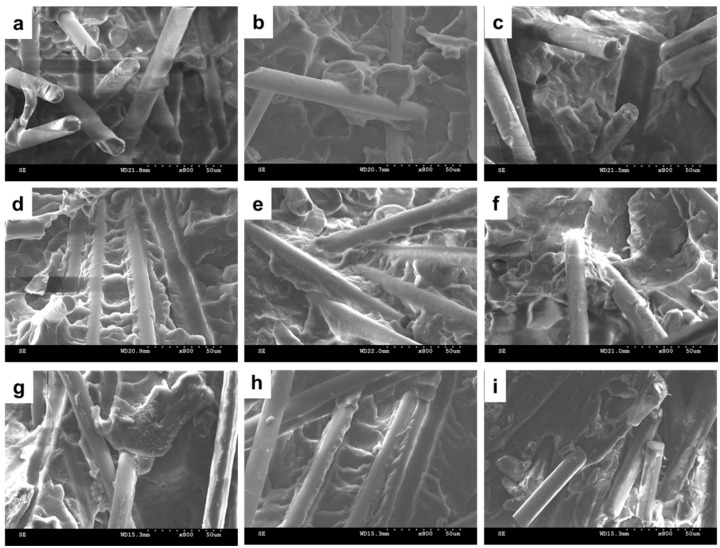
SEM images (800×) of PP/SGF composites, which are incorporated with (**a**) 0 wt % of a coupling agent; (**b**) 2 wt %; (**c**) 4 wt %; (**d**) 6 wt %; and (**e**) 8 wt % of PP-g-MA; as well as (**f**) 2 wt %; (**g**) 4 wt %; (**h**) 6 wt %; and (**i**) 8 wt % of SEBS-g-MA.

Figure 9 illustrates the SEM images of PP/SGF composites that are made with different amounts of coupling agent. The increasing amount of coupling agent, regardless of it being PP-g-MA or SEBS-g-MA, decreases the pull-out of SGF and the interstices between SGF and PP. In addition, the incorporation of a coupling agent also results in a rugged surface of PP/SGF composites. Therefore, PP-g-MA and SEBS-g-MA can effectively improve the interfacial compatibility between PP and SGF. Figure 10 shows the same images as those in Figure 9, but has a greater magnification. Figure 10 indicates that increasing PP-g-MA or SEBS-g-MA can distinctively decrease the interstices between SGF and PP. Meanwhile, the PP remarkably adheres to SGF, and SGF does not exhibit a pull-out phenomenon [30,34,35,37]. These results signify that the interfacial compatibility between PP and SGF has been improved, and the composites thus have greater mechanical properties, as exemplified in Figure 1, Figure 2 and Figure 3 and Table 2. These results confirm the findings of the study by Tjong *et al.* [34,35,37].

## 4. Conclusions

This study successfully improves the compatibility between PP and SGF for their composites by using PP-g-MA and SEBS-g-MA, and thereby augments the tensile strength, flexural strength, impact strength, thermal behavior, and compatibility. The test results have proven that SGF is a good reinforcing fiber, and the conjunction of 25 wt % of SGF improves the tensile, flexural, and impact strengths of PP. In addition, the incorporation of 8 wt % of PP-g-MA as a coupling agent provides the composites with 18% greater tensile strength, 24% greater flexural strength, and 33% higher impact strength; however, it does not benefit their tensile modulus or flexural modulus. Moreover, the incorporation of 8 wt % of SEBS-g-MA as a coupling agent provides the composites with 23% greater impact strength, but 25% lower tensile strength, 16% lower tensile modulus, 18% lower flexural strength, and 16% lower flexural modulus.

The test results also indicate that for PP/SGF composites, SGF serves as the nucleating agent, which increases the crystallization temperature of PP, but decreases the size of the spherulites of PP. Using PP-g-MA or SEBS-g-MA as the coupling agent allows for an even distribution of SGF, which at the same time provides more nucleating points for PP. As a result, the crystallization temperature of PP is increased and the spherulite size of PP is decreased. Hence, using these two coupling agents positively improves the interfacial compatibility, which is exemplified by the facts that the PP matrices enwrap the surface of SGF, and the mechanical properties of the PP/SGF composites are enhanced. The PP/SGF composites proposed by this study can also provide feasibilities for different practical applications by adjusting the amounts of PP-g-MA and SEBS-g-MA in order to render the composites with desired synthetic properties and diverse uses.
